# Genome-Wide Identification and Expression Analysis of *R2R3-MYB* Family Genes Associated with Petal Pigment Synthesis in *Liriodendron*

**DOI:** 10.3390/ijms222011291

**Published:** 2021-10-19

**Authors:** Lichun Yang, Huanhuan Liu, Ziyuan Hao, Yaxian Zong, Hui Xia, Yufang Shen, Huogen Li

**Affiliations:** Key Laboratory of Forest Genetics & Biotechnology of Ministry of Education, Co-Innovation Center for Sustainable Forestry in Southern China, College of Foresty, Nanjing Forestry University, Nanjing 210037, China; lcy@njfu.edu.cn (L.Y.); lhh91@njfu.edu.cn (H.L.); lxhzy1992@163.com (Z.H.); yxzong@njfu.edu.cn (Y.Z.); xia-hui625649@163.com (H.X.); shenyufang7045@163.com (Y.S.)

**Keywords:** *Liriodendron*, *R2R3-MYB* genes, pigment biosynthesis, petal coloration, RT-qPCR, HPLC

## Abstract

The MYB transcription factor family is one of the largest families in plants, and its members have various biological functions. *R2R3-MYB* genes are involved in the synthesis of pigments that yield petal colors. *Liriodendron* plants are widely cultivated as ornamental trees owing to their peculiar leaves, tulip-like flowers, and colorful petals. However, the mechanism underlying petal coloring in this species is unknown, and minimal information about *MYB* genes in *Liriodendron* is available. Herein, this study aimed to discern gene(s) involved in petal coloration in *Liriodendron* via genome-wide identification, HPLC, and RT-qPCR assays. In total, 204 *LcMYB* superfamily genes were identified in the *Liriodendron chinense* genome, and 85 *R2R3-MYB* genes were mapped onto 19 chromosomes. Chromosome 4 contained the most (10) *R2R3-MYB* genes, and chromosomes 14 and 16 contained the fewest (only one). MEME analysis showed that R2R3-MYB proteins in *L. chinense* were highly conserved and that their exon-intron structures varied. The HPLC results showed that three major carotenoids were uniformly distributed in the petals of *L. chinense*, while lycopene and β-carotene were concentrated in the orange band region in the petals of *Liriodendron tulipifera*. Furthermore, the expression profiles via RT-qPCR assays revealed that four *R2R3-MYB* genes were expressed at the highest levels at the S3P/S4P stage in *L. tulipifera*. This result combined with the HPLC results showed that these four *R2R3-MYB* genes might participate in carotenoid synthesis in the petals of *L. tulipifera*. This work laid a cornerstone for further functional characterization of *R2R3-MYB* genes in *Liriodendron* plants.

## 1. Introduction

MYB transcription factors (TFs) compose one of the largest superfamilies of TFs and exhibit various biological functions in plants. MYB proteins contain a conserved DNA-binding domain composed of one to four conserved repeats comprising approximately 50 amino acids per repeat. Accordingly, *MYB* superfamily genes are grouped into *MYB-related* (*1R-MYB*), *R2R3-MYB* (*2R-MYB*), *R1R2R3-MYB* (*3R-MYB*), and *4R-MYB* genes according to the number of imperfect MYB tandem repeats [1,2]. The R1R2R3-MYB family is the largest of the MYB superfamily in animals, while in plants, the R2R3-MYB family is the largest [3]. For example, there are 126 *R2R3-MYB* genes and 5 *R1R2R3-MYB* genes in *Arabidopsis thaliana* [4,5]. In plants, members of the MYB superfamily have various structures and functions, although the functions of most *MYB* genes are not clear. *MYB* superfamily genes have been identified in many plant species, including *A. thaliana*, moso bamboo (*Phyllostachys edulis*) [6], Chinese jujube (*Ziziphus jujuba*) [2], peach (*Prunus persica*) [7], and sesame (*Sesamum indicum*) [8]. There are also differences in the number of *MYB* genes identified in different plant genomes. For instance, there are 85 *MYB* genes in bamboo, 198 *MYB* genes in *A. thaliana*, 255 genes in peach, 171 genes in jujube, and 287 genes in sesame. With the identification of MYB superfamily members in many plant species, the functional study of MYBs has also become more in depth. Previous studies have shown that MYB TFs have three specific functions: the regulation of secondary metabolism, such as the biosynthesis of flavonoids [9]; the regulation of cell morphogenesis [10] and organ development, such as nectary development [11] and petal development [12]; and the mediation of signal transduction pathways in response to abiotic stress [13] and pathogen attack [14].

In plants, R2R3-MYBs constitute the largest TF family, and their members are involved in many biological processes, especially the synthesis of secondary metabolites. Pigments are important secondary metabolites involved in photosynthesis and petal coloration. Differential expression of *OgMYB1* was shown to modulate the pigmentation of floral organs in *Oncidium Gower* Ramsey [15]. Moreover, the novel gene *BrMYB2*, which regulates the accumulation of anthocyanins, was detected in the cultivar of purple head Chinese cabbage 11S91 [16].

*Liriodendron*, a tertiary relic genus, belongs to the Magnoliaceae family. At present, only two natural species of *Liriodendron* exist, *L. tulipifera* Linn and *L. chinense* (Hemsl.) Sarg. *L. tulipifera* is distributed throughout eastern North America, while *L. chinense* is scattered in southern China and northern Vietnam. *Liriodendron* plants are widely cultivated as ornamental trees because of their peculiar leaves, tulip-like flowers, and colorful petals. However, there is an obvious interspecies difference in petal color between *L. chinense* and *L. tulipifera*. The petals of *L. chinense* are dull and green in color, while those of *L. tulipifera* are colorful with an orange band in the middle. Petal color is one of the most important factors in relation to pollination. More colorful flowers are typically likely to attract more pollinators at a relatively long distance. Thus, petal color impacts the seed production and seed-setting rate of plants [12]. This notion partly explains why the seed-setting rate of *L. chinense* is lower than that of *L. tulipifera*.

Flower color is determined by the morphology of petal epidermal cells and the types of pigments [12,17]. Most angiosperms have conical epidermal cells. *MYB-related* genes are involved in the development of conical epidermal cells, thus enhancing the color intensity and brightness of petals [12]. The types of pigments generally include carotenoids, anthocyanins, and betalains [18]. A previous study reported that the synthesis and accumulation of carotenoids may play a key role in the formation of orange petal bands in *L. tulipifera* [17]. Nevertheless, these findings need to be verified through the use of a large amount of experimental data.

Gene family member identification, gene cloning, gene regulatory network reconstruction, and other functional analyses are largely dependent on genome information. Fortunately, an increasing number of plant genomes have been decoded in recent years, which has significantly accelerated the progress of plant genetics [19]. To date, only a small portion of *MYB* family genes have been identified in *L. chinense*. With the publication of *L. chinense* genome information [20], it has become possible to identify gene families from a genomic perspective. In this study, to test whether *R2R3-MYB* genes are involved in the accumulation of carotenoids or pigments synthesis during *L. tulipifera* floral development, we identified the R2R3-MYB gene family and revealed the precise spatiotemporal characteristics of orange band formation during *L. tulipifera* floral development based on the *L. chinense* genome and HPLC results. We found that the orange band pigments were lycopene and β-carotene, and identified four R2R3-MYB TFs might be involved in regulating the accumulation of carotenoids.

## 2. Results

### 2.1. Identification of Genes and Prediction of Encoded Proteins of the R2R3-MYB Family

Based on the annotated information of the *L. chinense* genome, we identified 255 potential *LcMYB* genes and obtained their corresponding protein sequences. The 255 protein sequences were subsequently screened for the presence of a MYB domain using the hidden Markov model (Pfam: PF00249), and 225 protein sequences containing a MYB functional domain were identified. To more accurately identify MYB family proteins, both Pfam and batch CD-search tests were performed for the 225 proteins. In total, 99 R2R3-MYB protein sequences were ultimately retained. Then, we predicted the physical and chemical properties of the 99 R2R3-MYB proteins, and the results are shown in Table S1. The molecular weight (*MW*) ranged from 15017.37 Da (Lchi25771) to 149250.3 Da (Lchi19398), and the isoelectric point (*pI*) ranged from 4.55 (Lchi08411) to 10.28 (Lchi29571). The average amino acid length, *MW*, and *pI* values were 319.83 aa, 35,891.72 Da, and 6.97, respectively. Among the 99 R2R3-MYB proteins, the isoelectric points of 62 proteins were less than 7.0. Thus, more than half of the R2R3-MYB proteins were acidic. Moreover, 85 *R2R3-MYB* genes were located across all 19 chromosomes; 14 genes were located on Contigs. Chromosome 4 had the most (10) *LcMYB* genes, and chromosomes 14 and 16 had the least (one) *LcMYB* genes.

### 2.2. Phylogenetic Analysis of R2R3-MYB Family Genes in L. chinense

**Figure 1:**
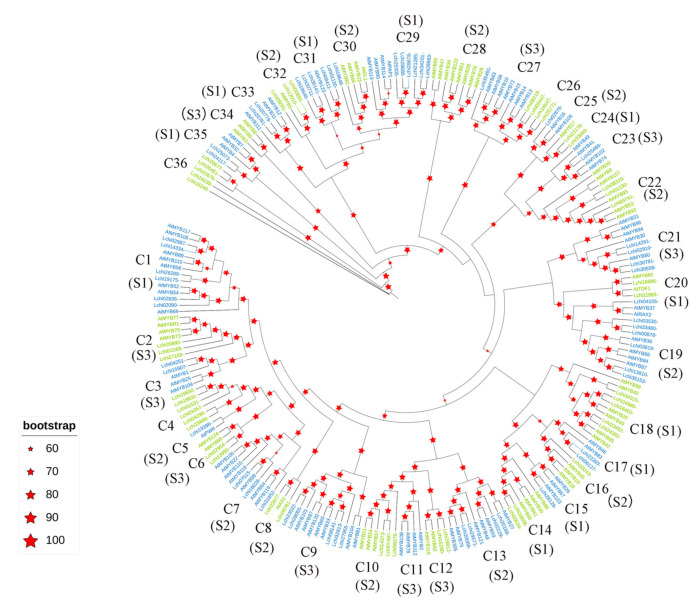
Phylogenetic tree of the R2R3-MYB family of *L. chinense* and *A. thaliana*. The phylogenetic tree was constructed based on 99 MYB proteins of *L. chinense* and 126 MYB proteins of *A. thaliana*. The red five-pointed star represents the bootstrap value. The larger the five-pointed star, the greater the bootstrap value.

A phylogenetic tree was constructed based on the 99 LcMYB proteins and 126 AtMYB proteins (Figure 1). The 99 *R2R3-MYB* family genes in *L. chinense* were classified according to the *R2R3-MYB* family genes in *A. thaliana*. The results showed that most of the subgroups were supported by strong bootstrap. Then, the phylogenetic tree was divided into 36 clades (designated C1–C36 in this study). The proteins were divided into the following three subgroups, named S1–S3, comprising 33 clades (Figure 1): S1: the regulation of secondary metabolism; S2: the regulation of cell morphogenesis and organ development; and S3: the mediation of signal transduction pathways in response to abiotic stress and pathogen attack. Among these three subgroups, the first subgroup (S1) includes C1, C14, C15, C17, C18, C20, C24, C29, C31, C33, and C35, and these clades are related to regulating the synthesis of lignin cellulose hemicellulose and secondary metabolites including procyanidins, anthocyanins [21], and flavonoids [22,23,24]. Because some AtMYB proteins with similar functions were clustered in the same clade, it may suggest that LcMYB proteins in the same clade had similar functions. According to the classification results, we selected 16 R2R3-MYB proteins for subsequent analysis. These proteins were located in clades C26, C29, C31, C33, and C35. These clades were related to pigment synthesis. Based on the classification results, LcMYB proteins may exhibit a wide range of functions and play an important role in the growth and development of *L. chinense*.

### 2.3. Gene Structure Analysis of R2R3-MYBs

**Figure 2:**
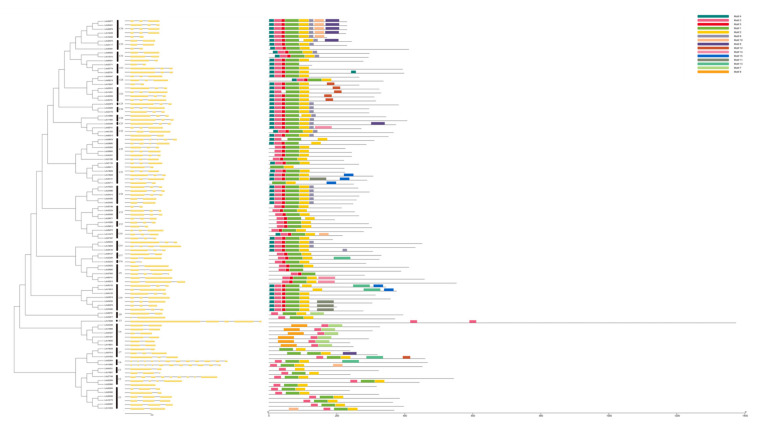
Phylogenetic relationship (**left**), exon/intron gene structures (**middle**), and motif distributions (**right**) of the *R2R3-MYB* family in *L. chinense*.

Exon/intron analysis showed that the coding regions of the 99 *R2R3-MYB* genes were interrupted by introns. The 99 genes were highly diverse in terms of their structure, including the number and relative positions of introns and exons. It is well known that gene structural diversity might be an explanation for the evolution of multigene families. A gene structure diagram of these 99 *R2R3-MYB* genes was constructed, demonstrating that the clustering pattern of the LcMYBs was not obviously consistent with the exon/intron structures (Figure 2). However, the genes of clade 31 exhibited similar exon/intron structures. This phenomenon is similar to that noted in sesame [8]. The number of exons ranged from 1 to 12, and was mainly 2 (32 genes) aprox 3 (56 genes) according to the *R2R3-MYB* gene structure. The Lchi19398 *R2R3-MYB* gene had the longest mRNA length and contained five exons. The Lchi20890 *R2R3-MYB* gene had only one exon, while the Lchi02954 gene had the most (twelve) exons. The relatively conserved structure of the small cluster genes also provides a basis for the classification of genes.

### 2.4. Conserved Motifs within R2R3-MYB Proteins

**Figure 3:**
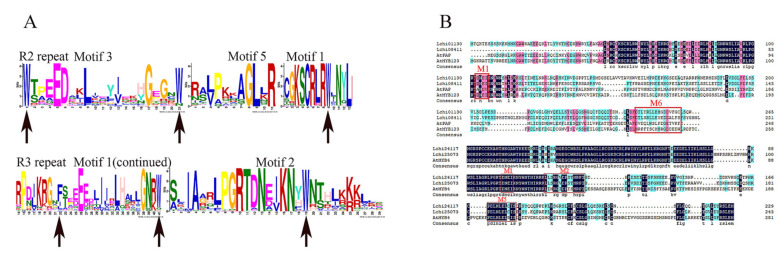
(**A**) R2 and R3 repeats of the proteins of the R2R3-MYB family in *L. chinense*. The R2 repeat and R3 repeat are composed of motif 3, motif 5, motif 1, and motif 2. (**B**) Characteristics of the amino acid sequences of candidate R2R3-MYB TFs in other species. Multiple sequence alignment was performed using DNAMAN. Four identified motifs are labeled M1, M2, M5, and M6. M1: NEDI/ANDV motif; M2: LIsrGIDPxT/SHRxI/L (C1-motif); M5: pdLNLD/ELxiG/S (C2-motif); M6: DX(D/E)S(X)3LL(D/N)(S/T)(D/E)(D/E)WP motif.

Fifteen motifs were predicted within the MYB protein sequences through the MEME online tool. Regarding the MYB protein motif types and sequence variations, the motif data sorting results were consistent with the phylogenetic tree results. Sixty-eight out of 99 R2R3-MYB proteins contained four highly conserved motifs, namely, motif 3, motif 5, motif 1, and motif 2, which contained 21, 11, 41, and 29 amino acids, respectively. The R2 repeat consists of motif 3, motif 5, and the first half of motif 1 (Figure 3A). The R3 repeat consists of the second half of the remaining protein of motif 1 and motif 2, while the R2 repeat contains a highly conserved isolated tryptophan (W) triplet needed to maintain its helix-turn-helix (HTH) structure with glycine (G) and leucine (L) at the eighth and ninth positions after the second W and nine conserved residues before the third W. These findings are consistent with findings in peach [7], sweet orange [25], and poplar [26]. Fifteen R2R3-MYB proteins contained three highly conserved motifs, namely, motif 3, motif 1, and motif 2, but not motif 5. The initial MEME analysis results showed that motif substitution existed in the R2R3-MYB family. Motif 4 was replaced by motif 8 in the new R2 repeat, whereas the R3 repeat was still composed of the remaining portion of motif 1 and motif 2 (Supplementary Figure S1). In the new R2 repeat, there are still three highly conserved W residues that form a new W triplet, and the position of W remains unchanged compared with that in the previous R2 repeat. The R2 and R3 repeats were also composed of highly conserved EED residues (glutamate (E)- E- aspartic acid (D)) and EEE residues (E- E- E). This phenomenon was also found in repeats of other plant species, such as poplar [26], tomato [27], Chinese white pear [28] and peach [7]. In group C15, six R2R3-MYB proteins contained three highly conserved motifs, namely, motif 8, motif 3, and motif 7, which contained 50, 21, and 49 amino acids, respectively.

The alignment of the amino acid sequences of LcMYBs and AtMYBs showed that all of these MYB TFs were conserved in their R2 repeat and R3 repeat, but more diversity was found in the *C*-terminal region. Lchi01130, Lchi08411, AtPAP1, and AtMYB123 (AtTT2) have M1 and M6 motifs [21,29,30]. Two AtMYB proteins, AtMYB3 and AtMYB4, acted as inhibitors of flavonoid synthesis. Lchi24117, Lchi25073, and AtMYB4 were in the same clade. These proteins had similar motifs, such as M1, M2, and M5 [30] (Figure 3B). This phenomenon suggested that these proteins may have similar functions in the biosynthesis of flavonoids and anthocyanins.

### 2.5. Dynamic Changes of Pigment during Petal Development

**Figure 4:**
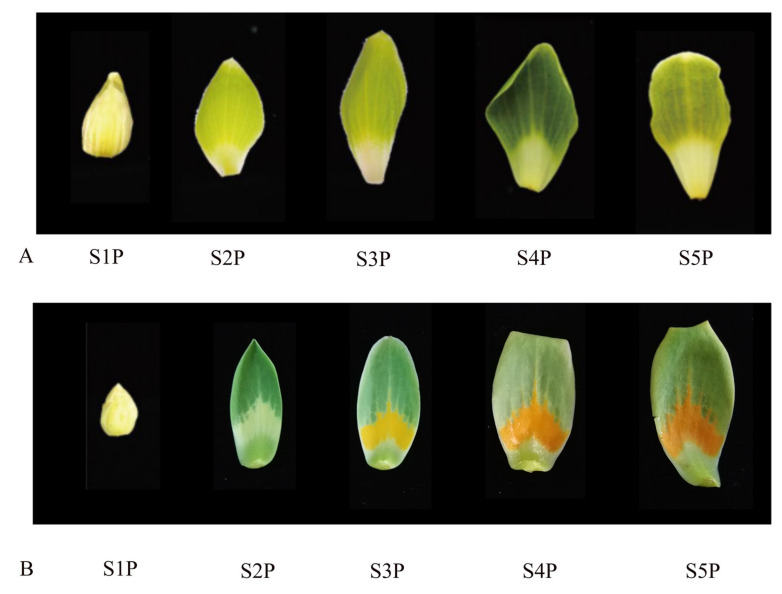
Petal samples at five stages in *Liriodendron*, ranging from S1P to S5P. S1P: all floral organs elongated; S2P: the petal was over twice the size of the petal in S1P; S3P: each petal of *L. tulipifera* present three colors obviously, with green on the top, yellow in the middle part, and white at base; S4P: the petal color of *L. tulipifera* changed from yellow to orange in the middle part; S5P: the petal color of the middle part deepened in *L. tulipifera*. (**A**) Five stages of *L. chinense* petals; (**B**) five stages of *L. tulipifera* petals.

**Figure 5:**
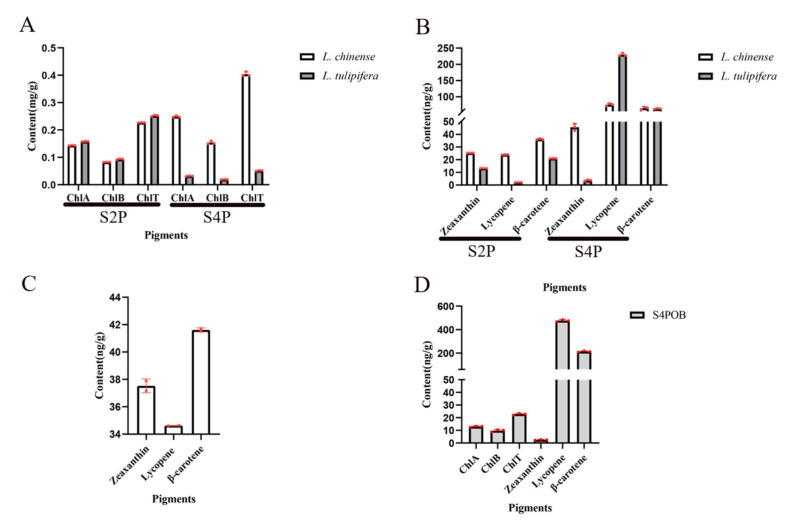
Pigment content of the petal samples. (**A**) Content of ChlA, ChlB, and ChlT. ChlA, ChlB, and ChlT represent the contents of chlorophyll A, chlorophyll B, and total chlorophyll, respectively. (**B**) The contents of β-carotene, lycopene and zeaxanthin. S2P represents the petal sample of *Liriodendron* at the S2P stage; S4P represents the petal sample of *Liriodendron* at the S4P stage. (**C**) The contents of β-carotene, lycopene, and zeaxanthin in the top region of *L. tulipifera* at the S4P stage. (**D**) The contents of ChlA, ChlB, ChlT, β-carotene, lycopene, and zeaxanthin in the orange band region of *L. tulipifera* at the S4P stage. Data indicate the mean ± SD and the dots represent raw data.

Only two natural species of *Liriodendron* exist: *L. tulipifera* and *L. chinense*. The petal color between these two species shows great divergence. To understand the changes pigments in the petals of *Liriodendron*, the pigments in petals were determined. The petal samples of S2P, S4P, the top region, and the orange band region in the S4P period were selected for chlorophyll and carotenoid determination (Figure 4). With the development of petals of *L. chinense* from the S2P stage to the S4P stage, the contents of chlorophyll A, chlorophyll B, and total chlorophyll increased significantly (Figure 5A). Surprisingly, the contents of chlorophyll A, chlorophyll B, and total chlorophyll decreased significantly during petal development of *L. tulipifera*. This phenomenon may be related to the petal coloring of *L. tulipifera*. From the S2P stage to the S4P stage, chlorophyll degradation occurred near the base (white region) of the petals of *L. tulipifera*, which was the first step in the formation of the orange band region [17]. According to the HPLC results, three carotenoids in *Liriodendron* were identified, including β-carotene, lycopene, and zeaxanthin. The content of these three carotenoids increased gradually during petal development in *L. chinense*. In contrast to the zeaxanthin content, the contents of the other two carotenoids also increased gradually during petal development in *L. tulipifera*. In conclusion, the total carotenoids of petals in *L. tulipifera* were higher than those in *L. chinense*, and carotenoids were evenly distributed in the petals of *L. chinense*. To further understand the pigment composition in the orange band region, we then divided the petals at the S4P stage into two regions (top region and orange band region) and determined the pigment. At the S4P stage, the content of zeaxanthin in the top part of the petals was approximately 15 times higher than that in the orange band region. The contents of β-carotene and lycopene in the orange band were approximately 5 times and 14 times higher than those in the top part of the petal, respectively. Therefore, we speculated that the orange band region contained mainly β-carotene and lycopene and that these two carotenoids in the petals of *L. tulipifera* were mainly concentrated in the orange band region.

### 2.6. Prediction of Cis-Acting Elements in the Promoters of 16 R2R3-MYB Genes

**Figure 6:**
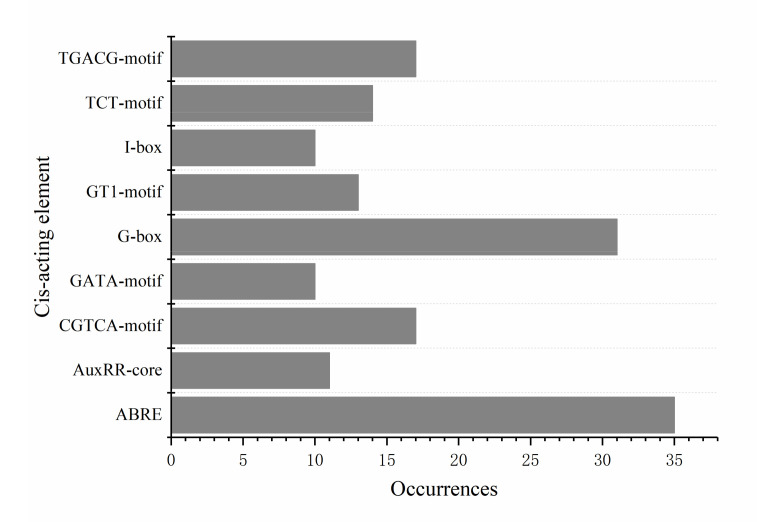
Occurrences of cis-acting elements associated with light and hormone responsiveness.

To identify cis-acting elements located within the 2000 bp upstream sequences of their promoters, 16 *R2R3-MYB* promoter sequences were obtained and analyzed using the PlantCARE online tool (Table S2). In total, 89 cis-acting elements were found in the promoters of the 16 *R2R3-MYB* genes. These cis-acting elements could be divided into five groups: light-responsive elements, biotic and abiotic stress elements, metabolism and development elements, hormone-responsive elements, and elements with an unknown function. The main elements associated with petal coloring were light-responsive elements and hormone-responsive elements, including the G-box (31 instances), the GT1-motif (13 instances), the TCT-motif (14 instances), the GATA-motif (10 instances), the I-box (10 instances), ABREs (35 instances), the AuxRR-core (11 instances), the CGTCA-motif (17 instances), and the TGACG-motif (17 instances) (Figure 6). The high occurrence of these cis-regulatory elements in the promoters of these 16 *R2R3-MYB* genes indicates that these genes likely play an important role in the petal-coloring process.

### 2.7. RT-qPCR Assays of Twelve R2R3-MYB Genes across Five Stages of Petal Development in Liriodendron

**Figure 7:**
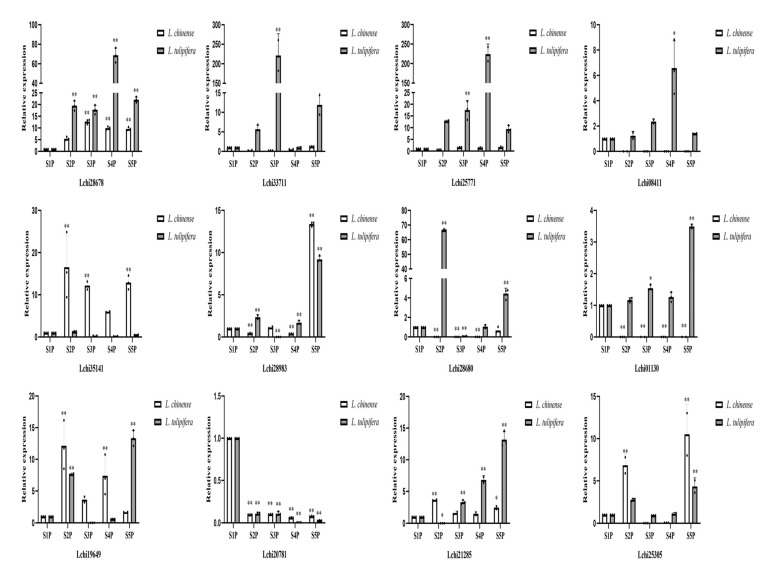
Relative expression of 12 *R2R3-MYB* genes in petals at five developmental stages. The details of the sequences of primers used are listed in Table S3. Data indicate the mean ± SD, and the dots represent raw data. **: *p* < 0.01, *: *p* < 0.05.

*R2R3-MYB* family genes participate in pigment synthesis in plants [31,32,33]. The expression profiles of genes in different tissues can provide references for the study of gene function. Based on our previous prediction of the function of the *R2R3-MYB* family genes, we ultimately selected 12 genes in both *L. chinense* and *L. tulipifera* that may be related to petal coloring and subjected them to RT-qPCR assays. Petals at five different developmental stages were collected from *L. chinense* and *L. tulipifera* (Figure 4). As shown in the expression profiles of 12 genes in *Liriodendron*, 10 genes exhibited different expression patterns (Figure 7). According to the RT-qPCR results, the expression profiles of the same gene in *L. tulipifera* and *L. chinense* were different. In *L. chinense*, the relative expression level of Lchi01130 was relatively high at the S1P stage and relatively low at the other four stages. However, the expression level of Lchi01130 at S1P-S4P was relatively stable, and the relative expression level was the highest at the S5P stage in *L. tulipifera*. The relative expression of Lchi35141 was relatively stable in *L. chinense*, but varied greatly in *L. tulipifera*. The relative expression levels of Lchi28680 in *L. chinense* were relatively low at each stage, but the expression levels in *L. tulipifera* were relatively high, especially at the S2P stage. The highest relative expression of Lchi21285 was recorded at the S2P stage in *L. chinense*, but the highest expression level was recorded at the S5P stage in *L. tulipifera*. Similarly, the highest expression level of Lchi19649 was recorded at the S2P stage in *L. chinense*, but the highest expression level was recorded at the S5P stage in *L. tulipifera.* The relative expression of Lchi28983 was highest at the S5P stage in both *L. chinense* and *L. tulipifera.* Four genes (Lchi28678, Lchi33711, Lchi25771, and Lchi08411) were highly expressed during the petal-coloring stages. Combined with the HPLC results and those of Hao et al. [17], we hypothesized that these four genes most likely participated in the formation of petal bands. In their study of the mechanism underlying basic orange band formation in *L. tulipifera* petals, Hao et al. [17] proposed that the expression of the genes most likely involved in orange band formation was upregulated and then downregulated, and the expression profiles of these four genes were consistent with their results.

## 3. Discussion

### 3.1. R2R3-MYB TFs Modulate Carotenoid Accumulation

Petal color is an important characteristic of flowering plants and is also the main factor that attracts pollinators. Compared with noncolorful petals, colorful petals can attract a greater number of pollinators, thus improving the seed-setting rate. Genetic modification of petal/fruit colors has been performed in kiwifruit [31], tomato [34], *Oncidium Gower* Ramsey [15], citrus [35], strawberry [36], orchids [37], and other plant species. Petal colors are determined by the nature of the pigment, the environment, and the morphology of petal epidermal cells [12,38]. The mechanism of petal pigmentation also differs in different plant species. Although the pathway of carotenoid synthesis is clear, little is known about the regulatory mechanism of carotenoid synthesis. The petal colors are significantly different between *L. chinense* and *L. tulipifera*. Moreover, the petal colors of *Liriodendron* hybrids are also different from those of its two parental species. Eleven quantitative characteristics of the flowers and fruits of *L. chinense*, *L. tulipifera*, and their interspecific hybrids (*L. chinense* × *L. tulipifera*) were determined and analyzed by Yu et al. [39]. In *Liriodendron* hybrids, β-carotene shows partial maternal and transgressive inheritance; thus, selecting *L. chinense* individuals with a high content of β-carotene as female parents would hopefully allow cultivation of *Liriodendron* hybrid progenies with orange petals [39]. Moreover, Luan et al. noted that the accumulation of carotenoids in different plant varieties/species is mostly due to changes in enzyme activity or gene expression [40], and numerous studies have indicated that the *R2R3-MYB* genes play an important role in petal fragmentation in many plants. Therefore, the identification of *R2R3-MYB* genes from the genome of *L. chinense* is of great significance for its subsequent breeding application.

At present, no clade related to carotenoid synthesis has been found in *A. thaliana* R2R3-MYB family genes, which also made it difficult to identify R2R3-MYB TFs related to carotenoid synthesis in other species. An R2R3-MYB TF *MtWP1* belonging to subgroup 6 was identified in an alfalfa flower color isolation population, and MtWP1 regulates carotenoid accumulation by combining MtTT8 and MtWD40-1 [41]. *MlRCP1* of R2R3-MYB subgroup 21 was identified to regulate carotenoid accumulation in *Mimulus lewisii* flowers [42]. Tomato *SlMYB72* directly binds to the *PSY*, *ZISO*, and *LCYB* genes and regulates carotenoid biosynthesis [43]. Phylogenetic analysis also resulted in the placement of *AdMYB7* in a clade together with *AtMYB112*, *AtMYB78*, and *AtMYB108*, but no homologous gene has been reported to be involved in the carotenoid biosynthesis pathway [31]. The R2R3-MYB TFs involved in the regulation of carotenoid synthesis in other plants were not grouped in the same clade of the phylogenetic tree. Therefore, we conjecture that other R2R3-MYB TFs of *L. chinense* that regulate carotenoid synthesis are present in other clades. Hao et al. determined the pigments of the orange band region of *L. tulipifera* in different developmental stages and their results showed that, as the contents of chlorophyll A, chlorophyll B, and total chlorophyll in the orange band region decreased, the content of total carotenoids increased with the deepening of the orange band [44]. The temporal pattern of pigment change in the orange band was consistent with that in the whole petal in *L. tulipifera*. The expression patterns of these four genes (Lchi33711, Lchi08411, Lchi25771, and Lchi28678) in *L. tulipifera* via RT-qPCR assays were consistent with the carotenoid accumulation trend in petals, indicating that these four genes may be involved in carotenoid synthesis in the orange band region. Whether these R2R3-MYB proteins bind to bHLH and WD40 proteins in the MBW protein complex needs to be further studied.

### 3.2. Supplement of R2R3-MYB Members and Functional Modules between L. chinense and A. thaliana

A total of 204 *MYB* superfamily genes were identified from the genome of *L. chinense* and were grouped into four families, including *1R-MYB* (99 genes), *R2R3-MYB* (99 genes), *R1R2R3-MYB* (5 genes), and *4R-MYB* (1 gene). Wu et al. identified 76 *R2R3-MYB* genes, but no *4R-MYB* genes from the genome of *L. chinense* [45]. Through the comparison of the original data, our data contained the 76 *R2R3-MYB* genes mentioned above. The Gene IDs of the 22 newly added *R2R3-MYB* genes are bolded in Table S1. The 99 *R2R3-MYB* genes we identified were further confirmed in multiple transcriptomes of *L. chinense.* The expression of the *99 R2R3-MYB* genes in different tissues of *L. chinense* is shown in Supplementary Figure S2; thus, our results are more reliable than the former, in which *R2R3-MYB* genes were identified from the genome of *L. chinense*.

To classify R2R3-MYB proteins in *L. chinense*, we used the R2R3-MYBs of *A. thaliana* as a reference. The LcMYBs were divided into 36 clades according to the phylogenetic and structural analyses. Then, we divided the 36 clades into three subgroups based on the function of the clades. The R2R3-MYB proteins in *A. thaliana* can be grouped into 32 clades, and some clades of R2R3-MYB proteins are species-specific in the phylogenetic tree. For example, there were only *A. thaliana* proteins in the C11, C28, and C34 clades. The functions of some members of these clades are related mainly to regulating jasmonate-mediated stamen maturation [46] and regulating indolic glucosinolate biosynthesis [47]. Similar findings were reported in *Beta vulgaris* [48] and soybean [49]. We identified members of clade C4 in *L. chinense* for which there are no representatives in *A. thaliana*, suggesting that these proteins might have specific functions that were either lost in *Arabidopsis* or differentiated at the time of the last common ancestor. Although the function of most LcMYBs remains unclear, the orthologs that clustered together into a functional clade shared a similar gene structure and functions, indicating a recent common evolutionary origin.

### 3.3. ABA Regulates the Accumulation of Pigments

Pigments exist not only in flowers and leaves with various colors, but also in the roots, seeds, and other parts of plants. The coloring process of fruits and petals is not only regulated by genetic factors, but also affected by external factors such as light, temperature, and hormones [50]. As one of the important hormones regulating plant growth and development, ABA plays a vital role in plant growth. As a key hormone in the process of ripening, ABA can significantly affect the accumulation of anthocyanins in plants. In Arabidopsis, anthocyanin synthesis pathway (ABP) genes are divided into two categories: early biosynthesis genes (EBGs; *CHS, CHI, F3H*) and late biosynthesis genes (LBGs; *DFR, LDOX, UFGT, LAR, ANS*) [51]. EBGs, leading to the production of flavonols, are activated by R2R3-MYB TFs, whereas the activation of the LBGs, leading to the production of pro-anthocyanidins and anthocyanins, requires a ternary complex composed of MYB-bHLH-WD40 (MBW) TFs [52,53,54]. After exogenous ABA hormone treatment, the expressions of *CHI*, *CHS*, and *MYB* genes and anthocyanin content in grapes were increased [55,56,57]. The same phenomenon also occurs in strawberries and sweet cherry [58,59].

Wang et al. speculated that ABA may reduce the expression of structural genes in the flavonoid pathway by regulating *CsMYB2* and *CsMYB26*, leading to a decrease in flavonoids [60]. To this day, it has been confirmed that exogenous ABA hormone regulates anthocyanin synthesis by affecting the expression of MYB TFs. Whether ABA affects the expression of bHLH, WD40, and the MBW complex remains to be further studied. Exogenous ABA affects not only the accumulation of anthocyanins, but also the accumulation of carotenoids and chlorophyll. Exogenous ABA significantly affected carotenoid metabolism in tomato, mango, watermelon, and citrus [61,62,63,64,65]. The expression of the *PSY3* and *DSM2* genes in maize and rice changed significantly after ABA treatment [66,67]. A previous study demonstrated that abiotic stress-induced ABA formation leads to the positive regulation of *PSY3* gene expression. Positive regulation increases PSY activity, feeding carotenoids into the pathway for the production of ABA [68]. In addition, Du et al. found that the activity of BC hydroxylases from rice, which were shown to be rate-limiting enzyme in terms of ABA biosynthesis, could alter plant resistance to drought stress by modulating the levels of xanthophylls and ABA synthesis [67]. The functions of *Arabidopsis* proteins clustered with these LcMYBs are mainly related to stress response and growth. For instance, three LcMYB proteins, Lchi27169, Lchi02269, and Lchi20890, were clustered with AtMYB70, AtMYB73, AtMYB77, and AtMYBR1/AtMYB44. Four genes, *AtMYB70*, *AtMYB73*, *AtMYB77*, and *AtMYBR1/AtMYB44*, showed obvious co-expression in *Arabidopsis* under a variety of abiotic stresses [69,70,71]. *AtMYB44* was highly expressed in the stomata of ducts and leaves in wild-type *Arabidopsis* treated with ABA, suggesting that *AtMYB44* may play a role in drought stress. The sensitivity of *AtMYB44* overexpression transgenic plants and knockout mutants to ABA and tolerance to drought and salt stress were enhanced, which further showed that *AtMYB44* plays an important role in the stress response [72]. High expression of *LchiMYB134* (Lchi20890) and *LchiMYB51* (Lchi02269) was detected in the quantitative *LcMYB* gene expression profile of 11 stages of *Liriodendron* hybrid somatic embryogenesis [45]; *LchiMYB134* was significantly upregulated under cold and heat stress, but significantly downregulated under drought stress. This indicated that *LchiMYB134* may play an important role in *Liriodendron* in response to abiotic stress. However, whether *LchiMYB134* increases ABA content through the carotenoid metabolic pathway to enhance plant drought resistance remains to be further studied.

The process of citrus fruit ripening is accompanied by the accumulation of carotenoids and the degradation of chlorophyll. Zhu et al. identified a R2R3-MYB TF that directly regulates the transformation of α- and β-branch carotenoids from a stay-green mutant of *Citrus reticulata* cv Suavissima [33]. Chlorophyll degradation and carotenoid accumulation are associated with the ripening of citrus fruits with a normal phenotype. In the sense of stay green phenotype, chlorophyll and carotenoids show accumulation. The EMSA results showed that *CrMYBb68* can regulate the expression of *NCED5* and reduce the ABA content [33].

The NCED enzyme is a key enzyme gene in the process of ABA synthesis [33,73]. Many transcription factors (*CrMYB68, WRKY57*, and *AtAF1*) control ABA biosynthesis by regulating the expression of *NCED* enzyme genes [33,74,75]. The low expression of *CrNCED5* in MT can explain the high content of ABA in MT-WT [33]. These results show that ABA is not only one of the metabolites of β-carotene, but also a feedback regulator of carotenoid metabolism. Our results show that the flower development of *L. tulipifera* is also accompanied by the degradation of chlorophyll and the accumulation of carotenoids [44]. Whereas, whether ABA participates in *L. tulipifera* petal coloring is still unclear, and needs to be uncovered furtherly.

## 4. Materials and Methods

### 4.1. Plant Materials

All the plant materials were collected from two adult trees in a provenance trial plantation of *Liriodendron* located at Xiashu Forest Farm, Jurong County, Jiangsu Province (119°13′ E, 32°7′ N) [76]. The *L. chinense* tree was originally collected from Wuyi Mountains, China, and the *L. tulipifera* tree was originally collected from Georgia, GA, USA. From March 2019 to early May 2020, we collected petals at five developmental stages (S1P, S2P, S3P, S4P, S5P) for RNA isolation. All plant materials were stored at −80 °C in a cryogenic refrigerator prior to RNA extraction.

### 4.2. Gene Identification and Coding Protein Prediction of L. chinense MYB Superfamily Members

The *L. chinense* genome sequence was downloaded from the National Center for Biotechnology Information (NCBI) database (https://www.ncbi.nlm.nih.gov/orffinder/?tdsourcetag=s_pcqq_aiomsg accessed on 21 March 2018, PRJNA418360.). Similarly, the MYB sequence data of *A. thaliana* was obtained from the PlantTFDB (http://planttfdb.cbi.pku.edu.cn/index.php accessed on 10 April 2020). The hidden Markov model (HMM) profile of the MYB-binding domain (PF00249) was obtained from the Pfam database (http://pfam.xfam.org/ accessed on 10 April 2020). The candidate MYB protein sequences of *L. chinense* were identified using HMMER tools, and the primary sequences were assessed using the NCBI Batch CD-search (https://www.ncbi.nlm.nih.gov/Structure/bwrpsb/bwrpsb.cgi accessed on 25 April 2020) and Pfam database to confirm the reliability of the candidates. The physical and chemical characteristics of the *MYB* superfamily genes identified from the *L. chinense* genome were predicted using the ProtParam online tool (https://web.expasy.org/protparam/ accessed on 5 May 2020).

### 4.3. Multiple Alignment and Phylogenetic Analysis

To group the R2R3-MYB proteins of *L. chinense*, we analyzed the phylogenetic relationships between *L. chinense* and *A. thaliana*. The phylogenetic tree comprised 99 R2R3-MYB proteins from *L. chinense* and 126 R2R3-MYB proteins from *A. thaliana*. We used MAFFT software (version.7, Research institute for Microbial Diseases, Osaka, Japan) to perform multiple comparisons of the protein sequences, and phylogenetic trees were constructed using IQ-TREE software (University of Vienna, Vienna, Austria) based on the maximum likelihood method. Bootstrap analysis with 1000 replicates was performed to calculate the reliability of the phylogenetic tree.

### 4.4. Gene Structure and Conserved Motif Analysis

Information on the 99 genes of the *R2R3-MYB* family was obtained from the *L. chinense* genome annotation data. The exon/intron structures of *LcMYB* genes were generated using the Gene Structure Display Server (GSDS) online tool [77] (http://gsds.cbi.pku.edu.cn/ accessed on 5 June 2020). The conserved motifs of the 99 LcMYB proteins were identified using the Multiple Em for Motif Elicitation (MEME) v5.1.1 online tool (https://meme-suite.org/meme/tools/meme accessed on 5 June 2020) [78]. The following parameter settings were used: motif discovery mode classic mode; sequence alphabet DNA, RNA, or protein; site distribution zero or one occurrence per sequence (zoops); and 15 motifs. The associated mast file was subsequently downloaded to visualize the MEME results using TBtools software [79].

### 4.5. Prediction of Cis-Acting Elements in the Promoters of Lcmybs

To identify the cis-acting elements present in the promoter sequences of the 16 *R2R3-MYB* genes of *L. chinense*, a 2000 bp sequence from the transcription start site (ATG) of the 16 *R2R3-MYB* genes was selected from the *L. chinense* genomic data using the PLANTCARE (https://bioinformatics.psb.ugent.be/webtools/plantcare/html/ accessed on 20 June 2020) online tool.

### 4.6. RNA Isolation and RT-qPCR Assays

Total RNA was extracted using TRIzol reagent (TIANGEN, China) following the manufacturer’s protocol. Residual genomic DNA was removed with DNase 1. RNA quality and concentration were measured via 1.0% agarose gel electrophoresis and a NanoDrop 2000 c spectrophotometer (Thermo Scientific, Wilmington, DE, USA). cDNA was synthesized using 500 ng of RNA and reverse transcription reagent (PrimeScriptTM RT Master Mix, TaKaRa, Dalian, China) in a 10 µL volume reaction solution. The cDNA used for RT-qPCR was extracted from flower tissues; therefore, the reference gene *LceIF3* was suitable (Supplementary Materials S1 Supplementary Methods) [80]. RT-qPCR assays were conducted on a StepOnePlusTM System (Applied Biosystems, Waltham, MA, USA) with a total volume of 10 µL per reaction. Each 10 µL reaction volume consisted of 10 ng of cDNA, 0.01 nmol of forward primers and reverse primers, 5 µL of 2 × SYBR Premix Ex Taq, and 0.2 µL of 50 × ROX reference dye (TaKaRa, Dalian, China). Gene expression was calculated using the 2^−ΔΔCT^ method with three technical replications.

### 4.7. Quantification of Photosynthetic Pigments and Carotenoids

Petal samples were ground under liquid nitrogen. A 0.2 g petal sample was used to extract pigment with 10 mL extracting solution. The extraction solution was composed of 20% (*v*/*v*) absolute alcohol and 80% (*v*/*v*) acetone [78]. The samples were placed in the dark and extracted at 37 °C for 2 days. The contents and types of carotenoids were detected by high-performance liquid chromatography (HPLC) instrument (Waters, UV2489, USA) coupled to an octadecyl silane (ODS) C18 analytical column (Supersil ODS2, 4.6 mm × 150 mm, 3 μm, Dalian, China) operated at 25 °C, with an injection volume of 20 µL and a flow rate of 1 mL/min. The mobile phase for HPLC consisted of acetonitrile (eluent A), ultrapure water (eluent B), methyl tertbutyl ether and methanol (V/V = 1:1, eluent C), and ethyl acetate (eluent D) [81]. The grading program was as follows: 0–6 min, 100% of eluent A; 7–12 min, 80% of eluent A, 10% of eluent B, and 10% of eluent D; 13–20 min, 65% of eluent A, 15% of eluent B, and 20% of eluent D; 21–22 min, 90% of eluent A and 10% of eluent D; 23–30 min, 50% of eluent A, 5% of eluent B, 40% of eluent C, and 5% of eluent D; 31–35 min, 30% of eluent A, 50% of eluent C, and 20% of eluent D; and 35–40 min, 100% eluent A. Carotenoids were detected at 450 nm and each sample had three biological replicates. The carotenoids contents were calculated by commercial pigment standards, including zeaxanthin, lycopene and β-carotene.

The contents of ChlA (chlorophyll A, mg·g^−1^ FW), ChlB (chlorophyll B, mg·g^−1^ FW), and ChlT (total chlorophyll, mg·g^−1^ FW) were detected in a UV spectrophotometer (UV-1600 (PC), AOE, Shanghai, China). The contents of ChlA, ChlB, and ChlT were determined using the following formulae, respectively:

ChlA = [(12.7 × *A* − 2.69 × *B*)/(*D* × 1000 × *W*)] × *V* (1)

ChlB = [(22.9 × *B* − 4.68 × *A*)/(*D* × 1000 × *W*)] × *V* (2)

ChlT = [(20.29 × *B* + 8.02 × *A*)/(*D* × 1000 × *W*)] × *V* (3)

where *A* is the absorbance at 663 nm, *B* is the absorbance at 647 nm, *D* is the distance travelled by the light path (0.8 cm), *W* is the weight of the petal samples taken (0.2 g), FW is fresh weight, and *V* is the volume of the extracting solution (10 mL).

### 4.8. Statistical Analysis

Statistically significant differences among various development stages of petals were determined using one-way analysis of variance (ANOVA) followed by LSD test for multiple comparisons. All statistical analyses were performed using IBM SPSS Statistics Version 22 software package (SPSS Inc., IBM Company Headquarters, Chicago, IL, USA). Data were presented as means ± standard deviation (SD).

## 5. Conclusions

In this study, 204 MYB TFs were identified in the genome of *L. chinense* and divided into four families: *1R-MYB* (99 genes), *R2R3-MYB* (99 genes), *R1R2R3-MYB* (5 genes), and *4R-MYB* (1 gene). We mapped 99 *R2R3-MYB* genes onto 19 chromosomes. Combining the HPLC and RT-qPCR results, we identified four *R2R3-MYB* genes (Lchi33711, Lchi08411, Lchi25771, and Lchi28678) that may be involved in carotenoid biosynthesis in *Liriodendron*.

## Data Availability

Not applicable.

## Supplementary Materials

The following are available online at https://www.mdpi.com/article/10.3390/ijms222011291/s1, Figure S1: Heat map of the 99 *R2R3-MYB* genes in different *L. chinense* tissues; Table S1: Physical and chemical property prediction, location, and exon number of the R2R3-MYB family in *L. chinense*; Table S2: Information on the 16 R2R3-MYB TFs; Table S3: Information of the primer sequences. Supplementary Materials S1 Supplementary Methods: The sequence of *LceIF3*.
