# Supplementary material for: Genome-Wide Identification and Expression Analysis of R2R3-MYB Family Genes Associated with Petal Pigment Synthesis in Liriodendron
Source: Int J Mol Sci. 2021 Oct 19;22(20):11291. doi: 10.3390/ijms222011291 (PMC8538729; doi:10.3390/ijms222011291)
Supplement: Supplementary file 1 [file ijms-22-11291-s001.zip › ijms-1379248-supplementary.pdf]

Supplementary materials

Table S1 Physical and chemical property prediction, location and exon number of the R2R3-MYB family in *L. chinense*

Gene ID	Amino acids (aa)	Molecular weight (*Mw*: Da)	Isoelectric point (*pI*)	No. exons	Location	Gene ID	Amino acids (aa)	Molecular weight (*Mw*: Da)	Isoelectric point (*pI*)	No. exons	Location
Lchi29571	194	22304.48	10.28	3	Chr1	Lchi01130	265	30209.03	7.09	3	Chr3
Lchi20781	398	44290.03	4.61	3	Chr2	Lchi16606	431	48119.95	7.17	4	Chr10
Lchi20779	394	43493.48	4.72	3	Chr10	Lchi09741	272	30827.16	7.2	3	Contig1978
Lchi24392	258	29059.76	5.07	2	Contig2089	Lchi34201	244	27671.34	7.64	3	Chr18
Lchi03357	204	23583.08	5.18	2	chr14	Lchi26289	385	43288.05	7.67	3	Chr1
Lchi16402	263	29872.92	5.23	3	Chr7	Lchi33480	278	30249.09	8.02	3	Chr1
Lchi25489	374	42157.02	5.35	3	Chr19	Lchi04105	314	35105.65	8.23	3	Chr15
Lchi04251	323	34845.93	5.38	2	Contig108	Lchi14373	217	24728.69	8.39	3	Chr5
Lchi06127	331	36918.83	5.43	2	Chr4	Lchi20676	230	25979.48	8.56	3	Chr2
Lchi27169	544	59738.54	5.47	9	Chr18	Lchi20677	230	25990.5	8.56	3	Chr12
Lchi19398	1374	149250.3	5.48	5	Chr18	Lchi20451	230	26004.61	8.56	3	Chr12
Lchi12010	293	32759.32	5.52	2	Chr9	Lchi24117	230	26178.79	8.61	2	Chr3
Lchi32229	253	29444.76	5.6	3	Chr8	Lchi02835	238	28041.27	8.65	3	chr12
Lchi33711	250	28119.06	5.62	2	Contig2389	Lchi35141	289	32279.04	8.7	3	Contig954
Lchi03619	358	40411.36	5.62	3	Chr15	Lchi04296	327	36781.33	8.71	2	Chr6
Lchi24986	296	33652.96	5.63	3	Contig2361	Lchi35153	345	38643.74	8.72	3	Chr17
Lchi30781	315	35108.18	5.67	3	Chr13	Lchi13810	376	42257.1	8.93	2	Chr16
Lchi13995	452	50534.56	5.72	11	Chr4	Lchi16239	227	25952.19	8.98	3	Chr7
Lchi01230	367	41132.35	5.82	3	Chr4	Lchi20068	265	30915.99	9.02	3	Chr11

**Gene ID**	**Amino acids (aa)**	**Molecular weight (*Mw*: Da)**	**Isoelectric point (*pI*)**	**No. exons**	**Location**	**Gene ID**	**Amino acids (aa)**	**Molecular weight (*Mw*: Da)**	**Isoelectric point (*pI*)**	**No. exons**	**Location**
Lchi22393	325	36408.2	5.89	2	Chr4	Lchi25073	244	27872.04	9.06	2	Contig2461
Lchi21285	221	25721.81	5.9	3	Chr7	Lchi20890	317	34767.35	9.08	1	Chr19
Lchi11983	407	45065.89	5.95	3	Contig398	Lchi15866	305	34600.58	9.1	2	Chr7
Lchi07515	320	36870.28	5.96	3	Chr2	Lchi28680	287	32509.37	9.28	4	Chr1
Lchi08219	337	38929.01	5.96	3	Contig2066	Lchi00878	200	22062.99	9.39	3	Chr15
Lchi02876	382	41914.61	6.09	3	Chr4	Lchi05001	278	31506.92	9.46	2	Chr7
Lchi00034	451	50166.84	6.1	3	Chr9	Lchi25305	225	26180.86	9.49	3	Chr7
Lchi30558	310	34851.09	6.12	3	Chr4	Lchi02269	443	49385.46	9.52	4	Chr4
Lchi14291	330	37071.28	6.12	3	Chr1	**Lchi16240**	171	19657.22	9.68	2	Chr2
Lchi08315	352	39400.1	6.18	2	Chr8	**Lchi35491**	412	46392.84	5.32	2	Contig519
Lchi33069	295	32373.1	6.19	3	Chr6	**Lchi08411**	222	24757.26	4.55	2	Chr2
Lchi23178	295	32401.16	6.19	3	Chr2	**Lchi02090**	324	36901.78	9.14	2	chr4
Lchi16280	294	33562.79	6.19	3	Chr5	**Lchi22877**	371	42650.68	9.15	2	Chr2
Lchi16028	239	27647.13	6.2	2	chr12	**Lchi25771**	127	15017.37	9.32	2	Chr1
Lchi16886	345	37727.35	6.21	3	Chr15	**Lchi08781**	395	44852.17	9.37	2	Chr6
Lchi05447	266	30280.07	6.32	3	Chr3	**Lchi32813**	552	61551.32	5.49	3	Contig1171
Lchi02910	325	36515.23	6.34	3	Chr4	**Lchi33243**	286	32232.96	5.5	2	Contig108
Lchi28129	306	34491.28	6.37	3	Chr8	**Lchi15907**	240	27001.21	5.86	3	Chr5
Lchi34315	266	30145.1	6.41	3	Chr11	**Lchi08141**	458	50747.65	5.87	2	Chr15
Lchi03530	304	33601.58	6.44	3	Chr1	**Lchi19831**	249	27873.01	6.31	2	Chr12
Lchi00655	296	33284.35	6.45	2	Contig1049	**Lchi19175**	365	40207.57	7.68	3	Chr17

Gene ID	Amino acids (aa)	Molecular weight (*Mw*: Da)	Isoelectric point (*pI*)	No. exons	Location	Gene ID	Amino acids (aa)	Molecular weight (*Mw*: Da)	Isoelectric point (*pI*)	No. exons	Location
Lchi27955	282	30629.55	6.51	4	Chr4	**Lchi28678**	310	35813.66	7.62	5	Chr12
Lchi07497	188	21707.28	6.52	3	Chr12	**Lchi02887**	397	44433.91	7.1	3	Chr2
Lchi19646	307	34370.77	6.54	3	Chr6	**Lchi16547**	266	30576.54	7.08	2	Contig1833
Lchi28065	389	42909.8	6.62	3	Chr7	**Lchi20188**	214	24899.1	6.91	2	Chr19
Lchi23492	248	27913.64	6.71	2	Chr11	**Lchi02954**	467	52278.8	6.74	12	Chr2
Lchi14334	369	40816.95	6.8	2	Chr11	**Lchi19830**	239	27262.52	6.61	2	Chr13
Lchi01531	294	31427.94	6.84	2	Chr10	**Lchi23022**	412	44777.88	6.37	2	Chr10
Lchi28983	244	27686.29	6.85	3	Chr19	**Lchi31852**	460	51268.46	6.35	3	Chr18
Lchi06375	279	31720.32	6.9	3	Chr9	**Lchi29812**	303	34461.57	6.34	2	chr18
Lchi19649	224	25268.43	6.97	3	Chr8						

Note: 99 *R2R3-MYB* genes were listed in Table S1, including 76 previously identified genes by wu et al and 22 new genes. Two different fonts were used to distinguish these two groups of genes. The Gene ID of 22 new genes was bolded in Table S1.

Table S2 Information on the 16 R2R3-MYB TFs

Gene ID	Clade	Gene ID	Clade
Lchi28678	C29	Lchi19649	C31
Lchi33711	C31	Lchi20781	C33
Lchi25771	C33	Lchi21285	C29
Lchi08411	C31	Lchi25305	C29
Lchi35141	C31	Lchi25073	C35
Lchi28983	C29	Lchi20779	C33
Lchi28680	C29	Lchi34201	C29
Lchi01130	C31	Lchi19646	C31

Note: Gene ID and clade information of 16 R2R3-MYB TFs used in *cis-acting* elements analysis.

Table S3 Information of the primer sequences

Gene ID	Primer sequences	Length/bp
EIF3-F	CATCCAATTTCACTTTCCGCTCCAC	25
EIF3-R	AATCACCAGCAGACGAGAAGCA	22
Lchi01130-FP	ACTTGCCGGAATCTGATCCAC	21
Lchi01130-RP	CCATGCTCCAGTAACCTCGT	20
Lchi08411-FP	CGTCGAAGTCCGTCTCAAGCA	21
Lchi08411-RP	ATTGGCAACAGATGGTCCCT	20
Lchi19649-FP	ACCCCTAATAAACCAAGTGTCCT	23
Lchi19649-RP	TCCCTCCGACACAACAAACCC	21
Lchi20781-FP	AGACTTTTCAACCAAGCCGCTCA	23
Lchi20781-RP	CCCCACTCTCTATCTCCTCACC	22
Lchi21285-FP	TCGCAGGTAGAATCCCAGGA	20
Lchi21285-RP	ACTCCATCTCGTCCACCTCG	20
Lchi25305-FP	CTAAGGGCAGGTCTCAAACGA	21
Lchi25305-RP	TTCTTCCCGGCAGTCTACCAG	21
Lchi25771-FP	TGGACAGCAGAAGAAGATCGGA	22
Lchi25771-RP	TCCCACACCGTAATAAGCCTGC	22
Lchi28678-FP	CATCAGCCTCGAACCTTCTCC	21
Lchi28678-RP	AGTCATTCCAATAGCACCCTCC	22
Lchi28680-FP	CAACCACAAGGCAGAGTTAGCA	22
Lchi28680-RP	CACCCCAAGCACTAACTCCAC	21
Lchi28983-FP	AAAGTCCTGAAGCCAAAGCCT	21
Lchi28983-RP	GCTCTTCCGTTGCATCTCCAC	21
Lchi33711-FP	TGCTTGCCCAATCCCCACA	19
Lchi33711-RP	GCGCCACGTAGACTTCTCCA	20
Lchi35141-FP	GCCCCTCCAGACAAAGATTCCT	22
Lchi35141-RP	AATCTTCCAGCATCCCCTCGT	21

Note: Gene ID and primer sequences of 12 R2R3-MYB TFs used in RT-qPCR assays.

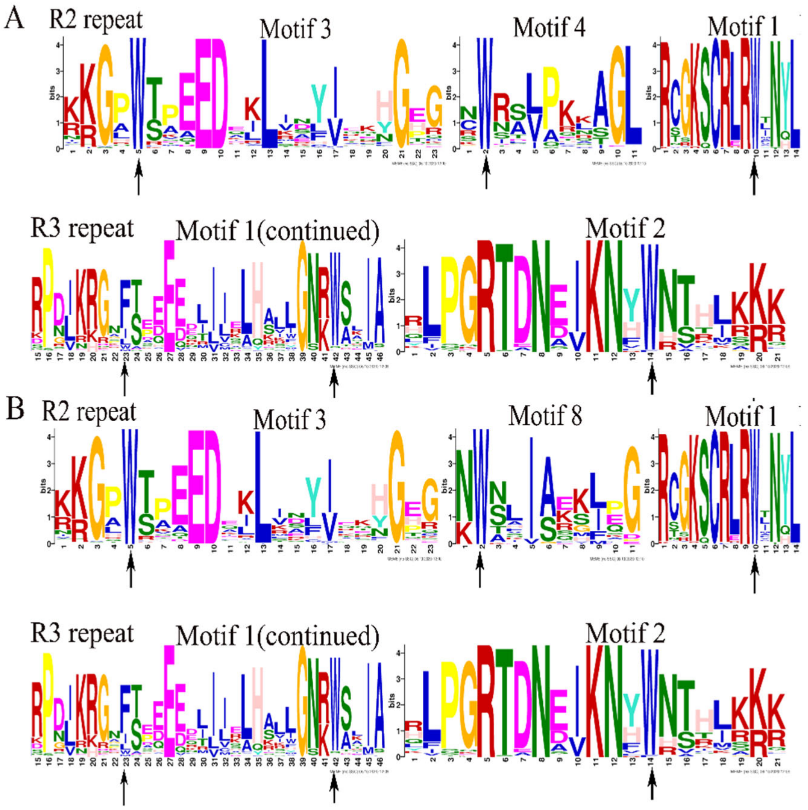

Supplementary Figure S1. Motif substitution in the R2 and R3 repeats. A: The R1 and R2 repeats of R2R3-MYB family proteins. The R2 repeat and R3 repeat are composed of motif 3, motif 4, motif 1 and motif 2. B: The new R1 and R2 repeats of R2R3-MYB family proteins. The new R2 repeat and R3 repeat are composed of motif 3, motif 8, motif 1 and motif 2.

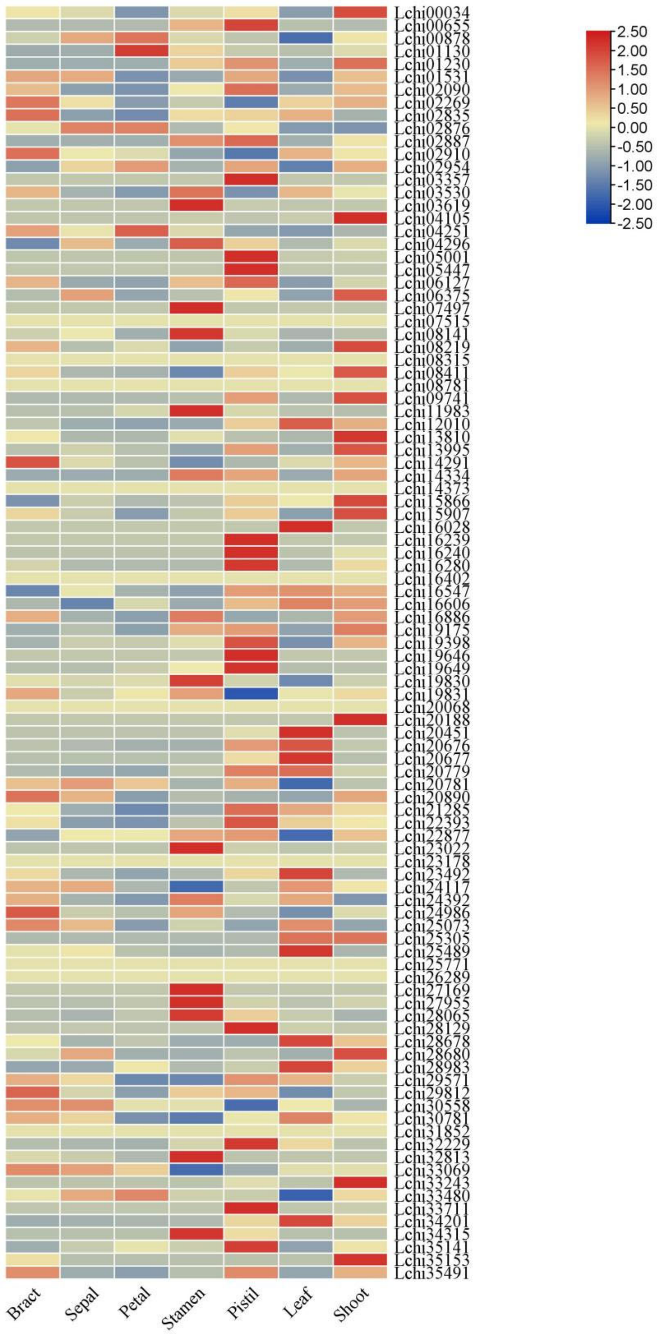

Supplementary Figure S2. Heat map of 99 *R2R3-MYB* genes in different *L. chinense* tissues. These tissues include bract, sepal, petal, stamen, pistil, leaf and shoot.

Appendix S1 Supplementary methods

The sequence of *LceIF3*

>LceIF3

TCTCCCCCTAGTTTTCCAAAGCTTGCAGGCAGAAATGAAAATAGTCCTCATGTAA
CCAAAACTGACTCTCTGCTAGTTTTTCAAGGGCTGAATCTCTTGTTTAGATAAATA
GAAAAAAGCAGCCAAGGTTCAACACACTGATGAAAAAAGAAAAATCAGTATTG
GACAAAAAAAAATTCCTCCACAAGGTTTACCTCTTAGAACATTCTCTTAGAACAC
ACCAACAGTTGATGCTTCAACAGGTGGGCCTCAGCGTTCGGATCCGGATGGAGG
AATTCTAGAAGAACCCCATGTGGATGACGAAGATCTAGAACCGCTGCCGCCAGA
AAACGCCGACTTGCGGTCGTCGCTACGCCACTTGTCACCAGAATGGGGCGGGCG
GTTGTCATGCTTGCCCCAACGGTCCTGTTCTGGAGGTGCGGCTGCACCCGAGCTT
TCCGTCTTTTCACGCCGGAACCTTGGCACATACTTTCCAGATGTGGGTGCTGCTG
CTGATGCCGCGACAGTGGCAGCCACAGGCTCCACGGGGCGGGGTCCATTGGCCT
GCTCGGCTGGCCGTGTGAGCGGCTCCGATGTCTTTCCTAAGAGAGCTTCTCTCCT
CAACCGCTCCTTCTCTTCCAATTCCTTCTCCCTCTGCCTCTGCCTCTCGGCGATCT
CATCCAATTTCACTTTCCGCTCCACCTCTTCCTTCTTGCGCCTTTCAGCCTCTTCGT
GCTTTCGAGCTTCTTCCTCTTCACGCAGCTTGGTCAGCCGCTCCTCCTCCGATTTT
ATAAAGAAAAGCAGTTTCCTTTTCTTCTCCCTTTCCTGCCTCCGCATAACTATGAG
CTGCCTGATCCTGTCCTCTCTCTCTTTCTTCAGTCTGTTATATTCTGCTTCTCGTCTG
CTGGTGATTCTATTATGCAATATATTCTTGTTGTCCAACATCCGGGCAAGCCGATT
TTTCTCTTGAAGGTCACCTGCATGATGCTGCCTGCTGAGCTCAATTTCTTGCAGCT
GTTCATGTTCATGGAGTATCTTCTCTTCCACCAGACGCTGTTGAAATGTTGTTGCG
ATCAAAGGTGCCTCCTCTTCTCTTTTGGCTCTTTCCATATAATCCATTGTTTTGGCA
AGTTTTTGCAATTTCTTCTCCATCTCCTGTCGCTCTCTGAGTTGTTCACTCAATGCC
AACTCAATCAAGGTCTGCTTTGTCACCTTTTCCCCCTCAATGACTGGCTTCTTTCC
CTTCTTCTTACGACCCTTCTCGGCTTCCTGAAGTAGAGCATGGGCCTCTTCAAGTT
CCCTCTCCTCTATTTCTCTGCGTATCCTCTGTTCTTCCCTTCTAGTATACTCAGAAG
CCAGTCTCTTTTGTTCTGCCTCCTCAGTAATTTTCTGTAGTTTTATCCTCTTTGACTC
TTCTTCCCGTTCCATTTCTAGCATCTGCCGTTCCTGTTCTTCCTTGCGTCTCTCAATT
ATCGATTTTCGGGCAAGTAGTCTTTTGTGCTCTCTATCCACAGTGTCTGCTAACAC
AGCTAGAGTATCACTTCCAAGCCGAGCTTGCAATGGAGGATAGATCATGCTCCTG
GCTTTGTTCAAAGATTCCGCAAAAATGGTAAGGTGATCCCGGAGCCTGTCGGAC
TCAAGATCCATACTACCAAAGAGAACAGCACCCTTCAAGTGGTCAACTTTCATG
GCAACAAAATTATATTTGACGGCATCTACTGAAATTTTCTCCACAACTGAGAAAT
CGAAAAAGGGAATCATCCTTGATATGACCTCGATTTTCATGGTTTGATAAACCTG
TGAAACCTGCTGTAGCACTCTCAAGGCAGCAAGTTTTTCCAGAGCAGGAACATA
CTGTGATAATTGCACTTCCGGAACAGAAGAAGCTGAAGAAATCTTGCCACCAAG
CTTAGAGATCTTGGACAACAACGGCTGAACTTTTGACACAAGATCTAATGGGAG
GAATTCATGTTCTAAAAGATGGAATAGGTCTTTTACTTCTTGTGAGACACATGTCA
ATACACCTTTGGAGGCCAATTCTGAGAGAAGTGATGATCGTGAAAGCTGCCAGA
GTTCAAGCTCTGTTGCAATCTTTAGCTGCTCAAATCTGGTATCTAAGTACAGCTGC
AAACTTTCTGGAGATGACAAGTCAGGTCTATCTCTTTGGTCTCTGTATTTGTTAAG
ATTAGCCAAGTGGTTTCGGATAATTTCACAAAGCCTCCGAAATTCTGTCGTCCGT

TTGTATTGCTTACAGAACTGGAAGGCCCGGTGTGCCGTCATCGCATATAAACCTT
CTAATTTTGAGTTGTTACGTAAAATTTCTAACACAGTCCTGTATGTCTCCCATAAA
AATTTGAACCATGGAGTTACAAGCTCCCGGTCAGACCTATCTCGACCTTTCTCAC
CACTGACATAGCTTAGCATCAAGTCTTCTGGCCTTTTGTCTGCTTCCAAATCATCA
ACATCCAAAGCATCTTCCAAGGCTTGCGCCTGGCTTCGAGCTTGCTCCGCCCTCT
CGGTGGAC

Note: The red labeled sequence is the forward primer position of RT-qPCR; The blue labeled sequence is the reverse primer position of RT-qPCR.
